# Validation and Application of an Ultra High-Performance Liquid Chromatography Tandem Mass Spectrometry Method for Yuanhuacine Determination in Rat Plasma after Pulmonary Administration: Pharmacokinetic Evaluation of a New Drug Delivery System

**DOI:** 10.3390/molecules21121733

**Published:** 2016-12-16

**Authors:** Man Li, Xiao Liu, Hao Cai, Zhichun Shen, Liu Xu, Weidong Li, Li Wu, Jinao Duan, Zhipeng Chen

**Affiliations:** 1 School of Pharmacy, Nanjing University of Chinese Medicine, Nanjing 210023, China; liman921005@163.com (M.L.); liuxiao04_0@163.com (X.L.); haocai_98@126.com (H.C.); 18260093567@163.com (Z.S.); m18260092677_1@163.com (L.X.); liweidong0801@163.com (W.L.); wuli87107@163.com (L.W.); dja@njutcm.edu.cn (J.D.); 2 Collaborative Innovation Center of Chinese Medicinal Resources Industrialization, Nanjing 210023, China

**Keywords:** yuanhuacine, pulmonary drug delivery, pharmacokinetics, ultra high-performance liquid chromatography tandem mass spectrometry

## Abstract

Yuanhuacine was found to have significant inhibitory activity against A-549 human lung cancer cells. However, there would be serious adverse toxicity effects after systemic administration of yuanhuacine, such as by oral and intravenous ways. In order to achieve better curative effect and to alleviate the adverse toxicity effects, we tried to deliver yuanhuacine directly into the lungs. Ultra high-performance liquid chromatography tandem mass spectrometry (UHPLC–MS/MS) was used to detect the analyte and IS. After extraction (ether:dichloromethane = 8:1), the analyte and IS were separated on a Waters BEH-C_18_ column (100 mm × 2.1 mm, 1.7 μm) under a 5 min gradient elution using a mixture of acetonitrile and 0.1% formic acid aqueous solution as mobile phase at a flow rate of 0.3 mL/min. ESI positive mode was chosen for detection. The method was fully validated for its selectivity, accuracy, precision, stability, matrix effect, and extraction recovery. This new method for yuanhuacine concentration determination in rat plasma was reliable and could be applied for its preclinical and clinical monitoring purpose.

## 1. Introduction

*Daphne genkwa Sieb. et Zucc.* (Thymelaeaceae) is a kind of traditional Chinese medicine (TCM), employed widely by Chinese people since ancient times. It has been used for the treatment of ascites, cough, asthma, and cancer in modern clinical applications [1]. Previous studies on medical chemistry showed that *D. genkwa* contains varities of chemical components, including flavonoids, diterpenoids, and coumarins [2,3,4,5]. Among them, daphne diterpene esters of yuanhuacina were discovered as an effective anticancer component [6]. In 2005, Zha-Jun Zhan et al. [7] reported the inhibitory activity of yuanhuacine against P-388 lymphocytic leukemia (IC_50_ = 30.0 μM), A-549 human lung cancer cells (IC_50_ = 0.15 μM), and endothelial cell HMEC (IC_50_ = 14.0 μM) in vitro, showing that it exhibits severely significant inhibitory activity against A-549 human lung cancer cells. Meanwhile, the anticancer mechanism of yuanhuacine has been found to be DNA topoisomerase I inhibitors by Zhang et al. [8].

The aim of our group was to apply yuanhuacine to curing lung cancer. However, it was reported that through the form of systemic administration, such as oral and intravenous ways, yuanhuacine can lead to liver, kidney, and reproductive system toxicity [9,10,11]. Usually, lung cancer occurs locally, so delivering the drug directly to the lesion area would be an effective treatment method. Based on our team’s previous studies, we planned to use the method of yuanhuacine dry powder inhalation. From the chemical structure of yuanhuacine, it could be concluded that the pulmonary epithelium is especially permeable. Physiologically, the whole lung has large areas for drug absorption [12]. Besides all of above advantages, enzymes have lower activity in the lung than in the other organs, which could be helpful for avoiding degradation caused by the first pass effect [13,14]. That is to say, the pulmonary delivery of drugs would be a viable alternative because of the attractive physiological properties of the lung. In all, pulmonary drug delivery could reach high drug doses rapidly and get directly to the site of disease while minimizing systemic toxicity compared with oral or intravenous dosing methods [15,16]. As for the pulmonary delivery of yuanhuacine, this mode of administration is brand-new. Therefore, it is necessary and essential to study the pharmacokinetics of yuanhuacine after pulmonary administration for further development of yuanhuacine relative preparations.

Present studies on yuanhuacine assay methodology are scarce. Yang et al. [17] used HPLC–UV to determine blood concentrations of yuanhuacine in rabbit plasma. However, it is difficult to apply this method to the detection of pulmonary delivery of yuanhuacine because of its comparably poor sensitivity. As we all know, it is necessary to monitor the blood concentration of poisonous drugs in clinic. Due to the low therapeutic dose of yuanhuacine for curing lung cancer, we need an effective assay method with a better sensitivity. That is why the UHPLC-MS/MS technique was applied as the golden rule for yuanhuacine quantification in this paper.

Zhang et al. [18] had tried to apply LC-MS/MS method into the determination of yuanhuacine in rabbit plasma. On the one hand, the mobile phase (acetonitrile–0.1% formic acid aqueous solution) in our method was simpler than that of their methodology, which was composed of methanol–acetonitrile–0.2% formic acid in water (85:5:10 *v*/*v*/*v*). Meanwhile, the retention time of yuanhuacine was shortened to 4.3 min compared with 9.9 min, representing that the analysis time was greatly shortened in order to avoid the waste of organic solvent. On the other hand, the lower limit of quantification (LLOQ) about yuanhuacine of their methodology was 10 ng/mL and the LLOQ in our method was 2 ng/mL. In comparison, our methodology showed higher sensitivity for biological samples of low concentration and fine availability for determining biological samples widely in vivo.

## 2. Results and Discussion

### 2.1. Optimization of the Experimental Condition

In this research, yuanhuacine gave stronger signal responses in positive ion mode than in negative ion mode. To get a good sensitivity, multiple reactions monitoring (MRM) was used for yuanhuacine quantification. The mass parameters of DP and CE were both optimized to enhance the ionization efficiency of yuanhuacine and IS. The DP for yuanhuacine and IS were 29.11 V and 43.29 V, and the CE for yuanhuacine and IS were 26.15 V and 26.06 V, respectively.

### 2.2. Method Validation

#### 2.2.1. Specificity

**Figure 1:**
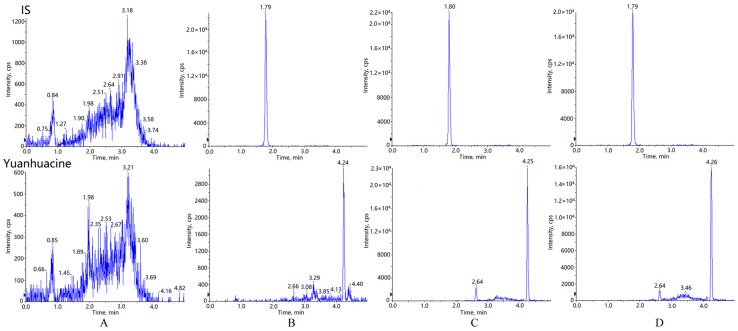
Representative MRM chromatograms for yuanhuacine and IS detection in plasma: (**A**) blank plasma; (**B**) blank plasma spiked with standard solutions in LLOQ; (**C**) plasma sample obtained 0.5 h after intravenous administration of yuanhuacine; (**D**) plasma sample obtained 0.5 h after pulmonary administration of yuanhuacine.

Typical chromatograms of blank plasma, blank plasma spiked with yuanhuacine plus IS solution at LLOQ, and plasma sample obtained 0.5 h after intravenous and pulmonary administration of yuanhuacine are presented in Figure 1. To exclude the interference of endogenous components or other impurities, blank samples from at least six rats were evaluated under the proposed extraction procedure and UHPLC–MS/MS conditions. The retention time of yuanhuacine and IS was 4.3 min and 1.8 min. The results of observation about the interference from endogenous substances in blank plasma were all acceptable.

#### 2.2.2. Linearity of Calibration Curves and LLOQ

The calibration curve was constructed by plotting the peak area ratios (yuanhuacine over IS, *y* axis), versus correspondent concentrations (*x* axis) within the concentration range of 2–500 ng/mL. The equation for the calibration curve was: *y* = 0.0234*x* − 0.0095. The correlation coefficient (r) was 0.9993. The LLOQ of yuanhuacine in plasma was 2 ng/mL. The precision and accuracy of LLOQ in this study were both within 5%.

#### 2.2.3. Precision and Accuracy

In this assay, the intra-day and inter-day precision/accuracy were determined by six replicate analysis of QC samples at three different concentration levels. The data were listed in Table 1. The intra-day and inter-day accuracy (RE) of Yuanhuacine was from −5.8% to 2.1%. The precision (RSD) was from 3.7% to 8.9%, both far less than 15%. All the obtained data were within their acceptable range, which indicated that the developed method was precise and accurate.

#### 2.2.4. Extraction Recovery and Matrix Effect

The extraction recovery and matrix effect of yuanhuacine were analyzed through three different standard concentrations. The recovery of yuanhuacine was evaluated by comparing peak areas of analyte in extracted samples with those in post-extracted spiked samples. The matrix effect was evaluated by comparing the peak area ratios of the analyte in post-extracted spiked samples with those of corresponding standard solutions. The results are shown in Table 2. The extraction recoveries of yuanhuacine at 5 ng/mL, 75 ng/mL and 400 ng/mL concentrations were 80.31% ± 3.33%, 77.86% ± 2.22% and 82.54% ± 4.54%, respectively. The recovery of IS was 89.63% ± 4.14%. The RSD of these data were all less than 12%. The matrix effects of drugs were within the range of 88.87%–103.7%. The matrix effect of IS was 95.28% ± 3.50%. It indicated that the matrix effects could be neglected.

#### 2.2.5. Sample Stability

The short-term stability was evaluated by exposing the samples at room temperature for 4 h. The long-term stability was evaluated by keeping the samples at the storage temperature (−20 °C) for 30 days. The freezing and thawing stability was assessed after three freeze-thaw cycles. The post-preparative stability was investigated in auto-sampler for 12 h after preparation. The results were in Table 3, indicating that the stability evaluation results were acceptable.

#### 2.2.6. Application of Pharmacokinetic Study

**Figure 2:**
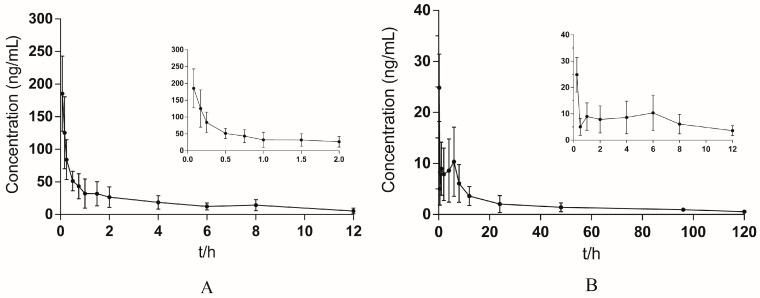
Mean plasma concentration-time curves of yuanhuacine after intravenous (**A**) and pulmonary (**B**) administration. The results revealed that the effects of yuanhuacine through pulmonary administration compared to intravenous injection could last for a longer time.

The validated method was successfully applied to the comparative pharmacokinetic studies of yuanhuacine in rats after intravenous and pulmonary administration. The mean concentration-time curves of yuanhuacine after intravenous and pulmonary administration are shown in Figure 2. DAS2.0 software has been used to calculate the pharmacokinetic parameters. The mean concentration-time curves about intravenous injection of yuanhuacine showed the highest correlation coefficient (r^2^ = 0.990) and the minimum Akaike’s Information Criterion (AIC = 73.276) by fitting on two-compartment model. Pulmonary drug delivery of yuanhuacine has the highest correlation coefficient (r^2^ = 0.997) and the minimum Akaike’s Information Criterion (AIC = 15.148) by fitting on one-compartment model. The pharmacokinetic parameters are shown in Table 4.

The area under the curve (AUC_0–t_) of yuanhuacine after pulmonary administration and after intravenous administration were 241.34 h∙ng/mL and 252.09 h∙ng/mL, respectively. Therefore, the absolute bioavailability (F) of yuanhuacine after pulmonary administration was 95.74% which directly reflects similar biological availability with that of intravenous administration. The biological half-life (t_1/2_) and the mean retention time (MRT) were 63.93 h and 65.38 h, representing that the effects of yuanhuacine through pulmonary administration could last for a longer time compared to intravenous injection. The difference of t_1/2_ between intravenous and pulmonary administration ways might be due to two main reasons. Firstly, pulmonary administration delivered the drug directly to the lung, in which way the local drug distribution was increased. However, the elimination of drug occurrence in the liver and kidney all depends on the carry work of blood circulation. As a result, pulmonary administration compared with intravenous administration leads to a slower absorption into blood circulation, so a slower elimination was expressed by half-life values. Secondly, part of the drug might be embedded in the pores of lactose, which also leads to a slow drug release.

At the same time, the C_max_ became stable and the T_max_ came later, indicating that blood concentration became more stable and time of yuanhuacine storage in rats also became longer after pulmonary administration. These changes could also help increase the compliance of yuanhuacine used in humans. That is to say, yuanhuacine used for pulmonary administration could decrease the toxicity by avoiding high concentration in blood. Study of the pharmacokinetic behavior of yuanhuacine inhalation provided the basic technical and theoretical information for the development of its new formulations.

## 3. Materials and Methods

### 3.1. Reagents and Materials

**Figure 3:**
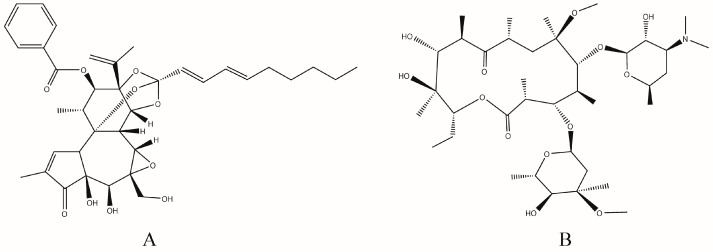
Chemical structures of yuanhuacine (**A**) and IS (**B**).

Yuanhuacine with a purity of 98% or higher was purchased from Shanghai Chinese Medicine Standardization Research Center (Shanghai, China). Clathromycin used as IS with purity of 98% or higher was purchased from Tokyo KaSei Industry Co., Ltd. (Tokyo, Japan). The structures of these two compounds are shown in Figure 3. Lactose was purchased from Meggle (Wasserburg, Germany). Pulmonary drug delivery device (DP-4M) was obtained from Penn-Century, Inc. (Philadelphia, PA, USA). HPLC-grade methanol and acetonitrile were purchased from E. Merck (Merck, Darmstadt, Germany). HPLC-grade water was obtained from a Milli-Q Reagent Water System (Billerica, MA, USA). Formic acid was of analytical grade and purchased from Nanjing Chemical Reagent Company (Nanjing, China).

### 3.2. Instruments and Conditions

#### 3.2.1. Instruments

The UHPLC–MS/MS system is composed of a Shimadzu UHPLC system equipped with a LC-30AD binary pump, an on-line degasser (DGU-20A5R), an autosampler (Model SIL-30SD), a column temperature controller compartment (CTO-30A), and a 5500 triple quadtandem mass spectrometer (AB Sciex, Concord, ON, USA) with an electrospray ionization (ESI) source. The separation of the analytes was achieved on a Waters BEH-C_18_ column (100 mm × 2.1 mm, 1.7 μm).

#### 3.2.2. UHPLC-MS/MS Conditions

**Figure 4:**
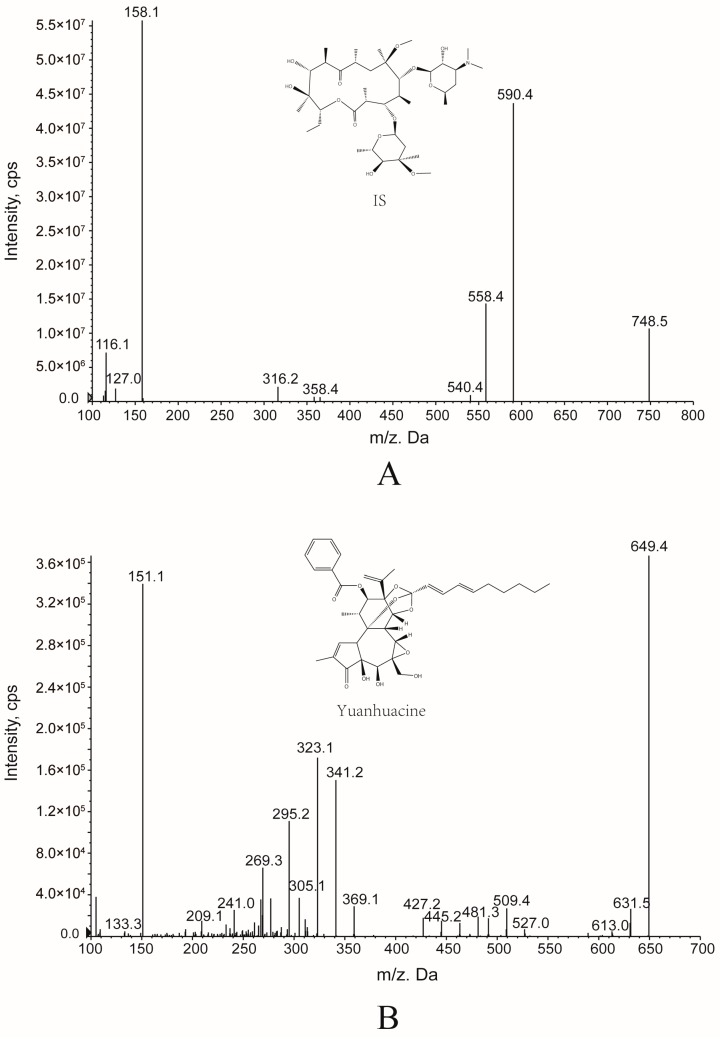
Mass spectra for IS (**A**) and yuanhuacine (**B**) detection.

The mobile phase was composed of a mixture of 0.1% formic acid aqueous solution (A) and acetonitrile (B) with a gradient elution program (0–0.1 min, 40% B; 0.1–2.5 min, 40%–95% B; 2.5–4.5 min, 95% B; 4.5–5.0 min, 95%–40% B).The flow rate was set at 0.3 mL/min and the injection volume was 5 μL. The ESI source was operated in positive ionization mode. The mass spectrometer was operated in multiple reactions monitoring (MRM) mode and the selected monitor ions were *m*/*z* 649.4/151.1 for yuanhuacine and *m*/*z* 748.5/590.4 for IS. The molecular ions in mass spectra for the analyte and I.S. were shown in Figure 4. The optimized parameters were as follows: ion source temperature (TEM), 550 °C; curtain gas (CUR), 35 psi; ion source gas 1 (GAS1), 55 psi; ion source gas 2 (GAS2), 55 psi; ion spray voltage (IS), 5500 V.

### 3.3. Animals

Ten male Sprague-Dawley rats, weighing 180–220 g, were supplied by the Slaccas Experiment Animal Company (Shanghai, China). The rats were kept in an environmentally controlled breeding room for five days at a temperature of 22–25 °C and a relative humidity of 50% ± 10% with food and water provided ad libitum. Before testing, animals were fasted overnight with free access to water. All animal experiments were carried out according to the Guidelines for the Care and Use of Laboratory Animals, and approved by the Animal Ethics Committee of Nanjing University of Chinese Medicine.

### 3.4. Preparation of Pulmonary Inhaled Powder Samples and Intravenous Injection Samples

An amount of yuanhuacine and lactose were mixed evenly (6 μg/mg) and used for yuanhuacine pulmonary inhalation. A certain amount of yuanhuacine dissolved in dimethyl sulfoxide (DMSO) and diluted with saline to 75 μg/mL was used for intravenous injection. The volume of DMSO in injection solution was 0.75%.

### 3.5. Preparation of Calibration Standards, Quality Control, and Internal Standard

Stock solutions of yuanhuacine (200 μg/mL) and IS (100 μg/mL) were prepared with methanol; The stock solutions were diluted into a serial of standard solutions. Calibration plots were constructed in the range of 2–500 ng/mL for yuanhuacine (2, 5, 25, 125, 250, and 500 ng/mL). The stock solution of IS was diluted with methanol to a concentration of 10 ng/mL. The calibration standards were prepared by spiking 90 μL blank plasma and 10 μL standard working solution. Quality control (QC) solutions (5, 75, and 400 ng/mL) of yuanhuacine were prepared in a similar way to get the calibration standards likewise. All of the stock solutions and QC samples were stored at 4 °C and warmed up to room temperature before being used.

### 3.6. Plasma Sample Preparation

To 100 μL plasma sample, 10 μL of I.S. solution and 400 μL of Na_2_CO_3_ solution (0.02 M) were added into an Eppendorf tube. The mixture was spiked with 1 mL extraction solvent (ether:dichloromethane = 8:1) and then eddied for 5 min of extraction. After centrifugation at 12,000 rpm for 5 min, the supernatant was then transferred into another Eppendorf tube and blown to dryness with nitrogen at 37 °C. The residue was reconstituted into 100 μL methanol, and centrifuged (12,000 rpm for 10 min). The supernatant of 5 μL was injected into UHPLC–MS/MS system for analysis.

### 3.7. Method Validation

Method validation was performed according to the FDA’s Guidance for Industry on Bioanalytical Method Validation [19].

#### 3.7.1. Selectivity

The selectivity was investigated by comparing the chromatograms of blank plasma obtained from six rats with those of corresponding standard plasma samples spiked with yuanhuacine and IS, as well as plasma samples after intravenous and pulmonary administration of yuanhuacine.

#### 3.7.2. Linearity and Lower Limit of Quantification (LLOQ)

The linearity of calibration curves was performed by the peak area ratios of yuanhuacine to IS in positive ionization mode versus plasma concentrations, and the calibration curves were constructed with a weight (1/*x*^2^) factor by least-square linear regression. The LLOQ was defined as the lowest concentration on the calibration curve at which an acceptable accuracy (RE) within ±20% and a precision (RSD) below 20% should be obtained.

#### 3.7.3. Precision and Accuracy

Six replicates of QC samples of analyte at three QC levels were included in each series to determine the intra- and inter-day (three days) precision of the assay. The concentration of each sample was calculated by the calibration curve each day. Accuracy was determined as the relative deviation in the calculated value of a standard from that of its true value. Precision was determined as the relative standard deviation (RSD) from the mean at each concentration level.

#### 3.7.4. Extraction Recovery and Matrix Effect

Extraction recovery of yuanhuacine was evaluated at three QC levels by comparing the peak areas of analyte in extracted samples with those in post-extracted spiked samples. Extraction recovery of IS was evaluated by comparing the peak areas of processed plasma samples with IS in post-extracted spiked samples. Matrix effect was evaluated by comparing the peak area ratios of the analyte in post-extracted spiked samples with those of the corresponding standard solutions.

#### 3.7.5. Stability

Stability of yuanhuacine in plasma were evaluated by analyzing QC samples of three levels during the sample storage and processing procedures. Short-term stability was evaluated by analyzing all QC samples kept at room temperature for 4 h before processing. Long-term stability was evaluated by analyzing all QC samples kept at −20 °C for 30 days before processing. Freeze-thaw stability was evaluated by analyzing all QC samples after three freeze-thaw (−20 °C to 25 °C) cycles. Post-preparation stability was assessed by analyzing the extracted QC samples kept in the autosampler at 4 °C for 12 h. All stability testing QC samples were determined using the calibration curve of the test day freshly prepared.

### 3.8. Pharmacokinetic Study

**Figure 5:**
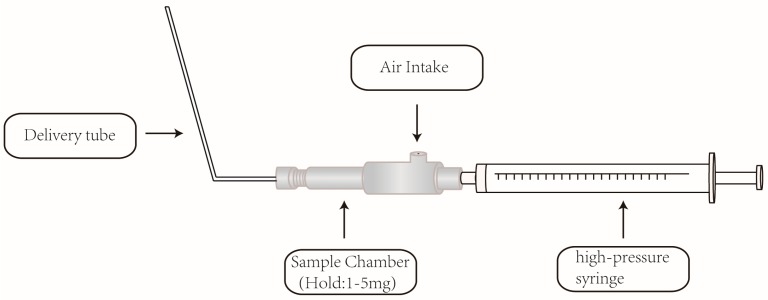
The schematic diagram of the pulmonary drug delivery device.

The rats were randomly divided into two groups, five for each. One group was administered in an intravenous way with yuanhuacine at a dose of 100 μg/kg. The blood samples (no more than 0.3 mL) were collected into 1.5 mL heparinized Eppendorf tubes at 0.08, 0.17, 0.25, 0.5, 0.75, 1, 1.5, 2, 4, 6, 8, and 12 h after dosing. Another group was pulmonary administration with yuanhuacine at the same dose. The blood samples (no more than 0.3 mL) were collected into 1.5 mL heparinized Eppendorf tubes at 0.25, 0.5, 1, 2, 4, 6, 8, 12, 24, 48, 96, and 120 h after dosing. The schematic diagram of pulmonary drug delivery device is shown in Figure 5. All the blood samples were immediately centrifuged at 12,000 rpm for 5 min to obtain plasma samples. The plasma samples were than stored at −20 °C until analysis.

## 4. Conclusions

In this study, a simple and rapid UHPLC-MS/MS method was developed to investigate the pharmacokinetic properties after yuanhuacine intravenous and pulmonary administration in rats. This method was then successfully used to monitor the drug concentration in rat plasma after pulmonary administration. This is the first research to study the pharmacokinetic properties of yuanhuacine after pulmonary administration. Also, pulmonary administration of yuanhuacine has been proven to be an effective way to increase curative effects and decrease toxicity. The results of this paper could provide theoretical basis for yuanhuacine preclinical and post-clinical application in the future.

## Figures and Tables

**Table 1:** Accuracy and precision of yuanhuacine determination in rat plasma (*n* = 6).

Compounds	Added Concentration (ng/mL)	Accuracy (%)		Presion (%)			
		Measured Concentration (ng/mL)	RE (%)	Intra-Day		Inter-Day	
				Measured Concentration (ng/mL)	RSD (%)	Measured Concentration (ng/mL)	RSD (%)
Yuanhuacine	5	4.71 ± 0.42	−5.8	4.71 ± 0.42	8.9	4.67 ± 0.36	7.8
	75	76.58 ± 3.92	2.1	76.58 ± 3.92	5.1	74.21 ± 4.93	6.6
	400	393.53 ± 14.45	−1.6	393.53 ± 14.45	3.7	401.94 ± 17.61	4.4

**Table 2:** The extraction recovery and matrix effect of yuanhuacine in rat plasma (*n* = 6).

Compounds	Added Concentration (ng/mL)	Extraction Recovery (%)	RSD (%)	Matrix Effect (%)	RSD (%)
Yuanhuacine	5	80.31 ± 3.33	4.1	93.47 ± 4.60	4.9
	75	77.86 ± 2.22	2.8	98.15 ± 5.11	5.2
	400	82.54 ± 4.54	5.5	99.69 ± 4.04	4.0

**Table 3:** Results of stability of yuanhuacine in rat plasma (*n* = 6).

Storage Condition	Added Concentration (ng/mL)	Measured Concentration (ng/mL)	RE (%)	RSD (%)
Short-term stability	5	4.77 ± 0.14	−4.6	2.9
	75	74.38 ± 2.60	−0.8	3.5
	400	395.34 ± 13.05	−1.2	3.3
Long-term stability	5	4.75 ± 0.40	−5.0	8.3
	75	74.77 ± 4.41	−0.3	5.9
	400	399.14 ± 14.22	−0.2	3.6
Freezing and thawing stability	5	4.71 ± 0.37	−5.8	7.9
	75	74.37 ± 1.80	−0.8	2.4
	400	392.62 ± 9.94	−1.8	2.5
Post-preparative stability	5	4.77 ± 0.32	−4.6	6.8
	75	74.36 ± 4.94	−0.8	6.6
	400	397.82 ± 8.91	−0.5	2.2

**Table 4:** Pharmacokinetic parameters of yuanhuacine in rats after intravenous and pulmonary administration (*n* = 5).

Parameters	Intravenous Administration of Yuanhuacine	Pulmonary Administration of Yuanhuacine
AUC_0–t_ (h∙ng/mL)	252.09 ± 103.70	241.34 ± 106.53
AUC_0–∞_ (h∙ng/mL)	308.94 ± 133.42	302.22 ± 141.22
MRT_0–t_ (h)	3.48 ± 0.52	35.38 ± 6.93
MRT_0–∞_ (h)	5.83 ± 2.61	65.38 ± 19.41
t_1/2_ (h)	5.28 ± 1.95	63.93 ± 33.08
C_max_ (ng/mL)	185.35 ± 57.71	24.86 ± 6.59
T_max_ (h)	0.08	0.25
